# All-Optical Rapid Formation, Transport, and Sustenance of a Sessile Droplet in a Two-Dimensional Slit with Few-Micrometer Separation

**DOI:** 10.3390/mi14071460

**Published:** 2023-07-21

**Authors:** Yuka Takamatsu, Chizuru Yamato, Masashi Kuwahara, Yuta Saito, Toshiharu Saiki

**Affiliations:** 1 Graduate School of Science and Technology, Keio University, Yokohama 223-8522, Kanagawa, Japan; 2 National Institute of Advanced Industrial Science and Technology, Tsukuba 305-8560, Ibaraki, Japan

**Keywords:** sessile droplet, laser heating, solutocapillary Marangoni flow, binary mixture liquid, phase-change material

## Abstract

We present a sessile droplet manipulation platform that enables the formation and transport of a droplet on a light-absorbing surface via local laser-beam irradiation. The mechanism relies on solutocapillary Marangoni flow arising from a concentration gradient in a binary mixture liquid. Because the mixture is strongly confined in a two-dimensional slit with a spacing of a few micrometers, the wetting film is stably sustained, enabling the rapid formation, deformation, and transport of a sessile droplet. In addition, to sustain the droplet in the absence of laser irradiation, we developed a method to bridge the droplet between the top and bottom walls of the slit. The bridge is stably sustained because of the hydrophilicity of the slit wall. Splitting and merging of the droplet bridges are also demonstrated.

## 1. Introduction

The nondestructive optical manipulation of microscale-to-nanoscale objects is a powerful technique in fluidic devices for biochemical and biophysical applications [1,2,3,4,5]. One example is the isolation and sorting of biological cells in microfluidic devices for cell-based high-throughput assays [6,7,8,9]. One of the common techniques for optical manipulation is optical tweezing, in which a tightly focused laser beam is used to trap microscopic objects by the restoring force arising from steep light-intensity gradients near the focal point. Optical tweezers have been intensively studied as a versatile tool [10]; however, they generally use a high-intensity laser beam that can damage sensitive samples.

As an alternative approach, droplet-based encapsulation and manipulation of single objects have been rapidly developed for high-throughput digital analysis on reconfigurable platforms [11,12,13,14,15,16,17]. For optical manipulation of a sessile microdroplet, optoelectronic mechanisms on functionalized substrates have been proposed and experimentally demonstrated. One of the successful implementations of this mechanism is the so-called optoelectrowetting device, which enables the surface wettability of the substrate to be locally modulated by light illumination [18]. Although the device operates at low light intensity, its structure is complex, comprising thousands of electrodes connected to a common bias through photoconductive wires. Recently, another optoelectronic approach based on the photovoltaic effect in ferroelectric materials (e.g., LiNbO_3_) was demonstrated [19,20,21]. In the case of photovoltaic optoelectronic tweezers, the directional excitation of electrons by light illumination generates a bulk electric field that extends outside the crystal to affect a droplet. The method does not involve electrodes or a power supply and enables operation under low light power. Trapping and transport of droplets have been realized; however, the creation of a droplet from a reservoir is less controllable. In addition, LiNbO_3_ is too expensive to be used as a disposable material.

The optical formation and manipulation of a sessile droplet has also been demonstrated using the solutocapillary Marangoni effect in a binary mixture of liquids, one of which has a higher surface tension and a lower vapor pressure than the other [22,23,24,25]. When a laser beam heats a layer of the mixture, the volatile component evaporates, causing solutocapillary flow toward the irradiated area. The capillary flows lead to an accumulation of the mixture, and a droplet is eventually formed in the irradiated area. In previous studies, Ivanova et al. started with a layer of a mixture containing dye molecules to enable absorption of light and created a droplet in a two-step process: thinning the layer through thermocapillary deformation, followed by the aforementioned droplet formation process [22,23,24,25]. Although they demonstrated remarkable control of droplet size by modulating the laser power, the growth rate of the droplets and their transport speed were inferior to those achieved via an optoelectronic mechanism. This inadequate growth rate and transport speed may arise from an excessively long thinning and accumulation time for the mixed layer as a result of the high initial thickness of the binary mixture. Furthermore, contamination by doping of light-absorbing substances is unfavorable.

In the present study, to achieve rapid growth and movement of a droplet, we propose the optical formation of a sessile droplet from a much thinner wetting film (<0.2 μm) of a binary mixture compared to those in the previous studies (~100 μm) [22,23,24], thus allowing to skip the aforementioned first step of droplet formation. The wetting film was formed by the recession of the mixture surface due to evaporation and was sustained by confining the mixture between two parallel plates with micrometer-scale separation. One of the plates was coated with a light-absorbing layer to enable the mixture to be heated without relying on doping. We achieved substantial improvements in the growth rate and transport speed of the droplet. More importantly, we demonstrated the sustainable formation of a droplet by bridging it between two parallel plates.

## 2. Materials and Methods

**Figure 1:**
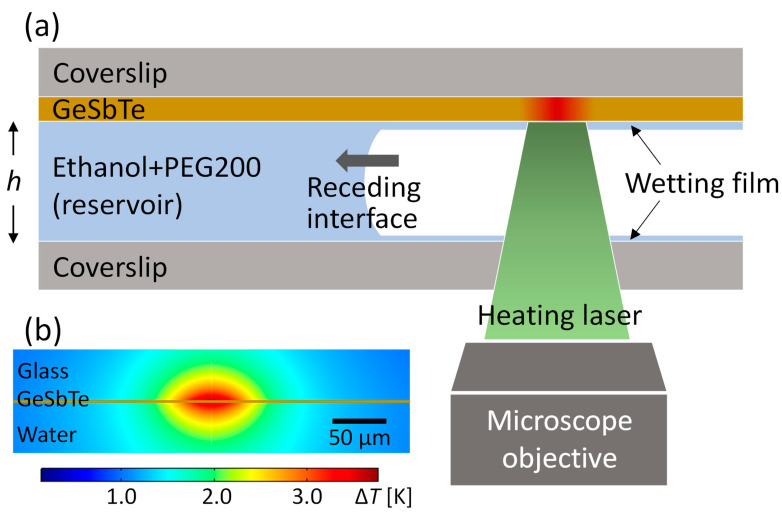
(**a**) Schematic of the experimental setup; h is the separation between two coverslips. (**b**) Cross-sectional temperature distribution profile for laser heating with a spot diameter of 50 μm when the incident laser power is 0.48 mW.

To demonstrate the proposed method, we used a binary mixture of ethanol and polyethylene glycol with a molecular weight of 200 (PEG200) with a volume ratio of 7:1. Ethanol served as the volatile liquid (vapor pressure *p* = 5600 Pa) with a surface tension of *γ* = 22 mN/m, whereas PEG200 was nonvolatile (*p* = 1.3 Pa and *γ* = 44 mN/m). Figure 1a illustrates the experimental setup for light-driven microdroplet formation, transport, and sustenance. The mixture was sandwiched between two glass coverslips with a separation of 2 ≤ *h* ≤ 4 μm, which was measured in every experiment. A wetting film was spontaneously formed as the mixture–vapor interface receded by evaporation. The confinement of the mixture to such a small-height channel contributed to the rapid formation and transport of droplets by maintaining a high vapor pressure in the channel and by accelerating the evaporation and condensation cycle.

As a light-absorbing layer, amorphous Ge_2_Sb_2_Te_5_ (GST) with a thickness of 100 nm was deposited on the top coverslip by radiofrequency magnetron sputtering. GST is widely used in storage media, including rewritable optical discs [26], and has a large absorption coefficient [27] and low thermal conductivity [28], both of which are advantageous for generating a steep temperature gradient in the experimental demonstration of our proposed method. A continuous-wave diode-pumped solid-state laser with a wavelength of 532 nm was used to heat the GST layer. The divergence of the laser beam before it was incident on the microscope objective was adjusted to increase the illumination diameter to 50 μm. Figure 1b shows a cross-sectional temperature increase profile, with a laser power of 0.48 mW, simulated using COMSOL Multiphysics. The reflected laser beam was rejected by a long-pass filter before the droplet dynamics were captured using a CMOS camera (Hamamatsu Photonics, Hmamatsu, Japan) with a capture rate of 50 frames per second.

## 3. Results and Discussion

**Figure 2:**
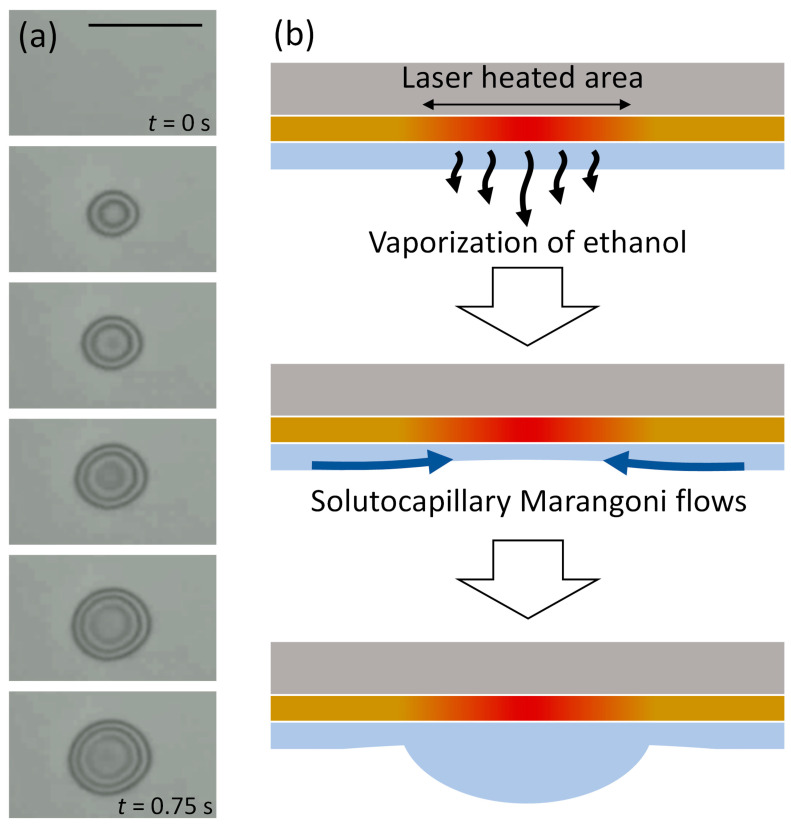
(**a**) Sequential snapshots of droplet formation process with a laser power of 0.48 mW. Snapshots were captured every 0.15 s. The scale bar is 50 μm. (**b**) Schematic of droplet formation in a wetting film of a binary mixed liquid under laser irradiation.

Figure 2a shows snapshots from a video recorded during droplet growth under laser irradiation with a power of 0.48 mW. A sessile droplet was rapidly formed immediately after laser irradiation started, and the footprint diameter (base diameter *D*_B_) was saturated within 0.6 s (Video S1). The mechanism of droplet formation can be explained as follows (Figure 2b): (i) the wetting film of a binary mixture is heated by laser irradiation, and the volatile component (ethanol) is vaporized according to the laser intensity profile; (ii) the vaporization leads to an increase in concentration of the nonvolatile and larger-surface-tension component (PEG200), causing a surface-tension gradient and a solutocapillary flow in the depth direction of the wetting film toward the center; (iii) the solutocapillary flow leads to the accumulation of the mixed liquid to form a sessile droplet, which can be maintained in stable equilibrium with its vapor during laser irradiation.

**Figure 3:**
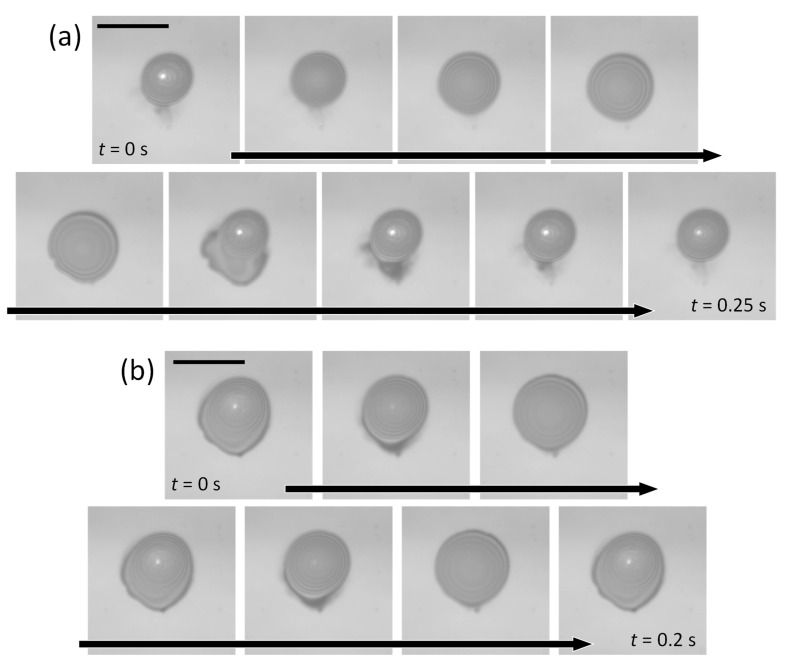
Sequential snapshots of the droplet deformation process by modulating the laser power with a mechanical chopper: (**a**) one modulation cycle at 4 Hz; (**b**) two modulation cycles at 10 Hz. The scale bar is 50 μm.

To examine the dynamic response time in droplet deformation, the laser beam was modulated with a mechanical chopper, and dynamical changes in the droplet shape were observed. Figure 3a,b show the time sequence of droplet deformation at modulation frequencies of 4 and 10 Hz, respectively. The repetitive accumulation and release of the mixed liquid from and toward the surrounding wetting film were clearly visualized at a modulation frequency of 4 Hz (Video S2). Even at 10 Hz, a 10% change in the footprint diameter of the droplet was observed (Video S3). No modification was observed at 30 Hz modulation.

**Figure 4:**
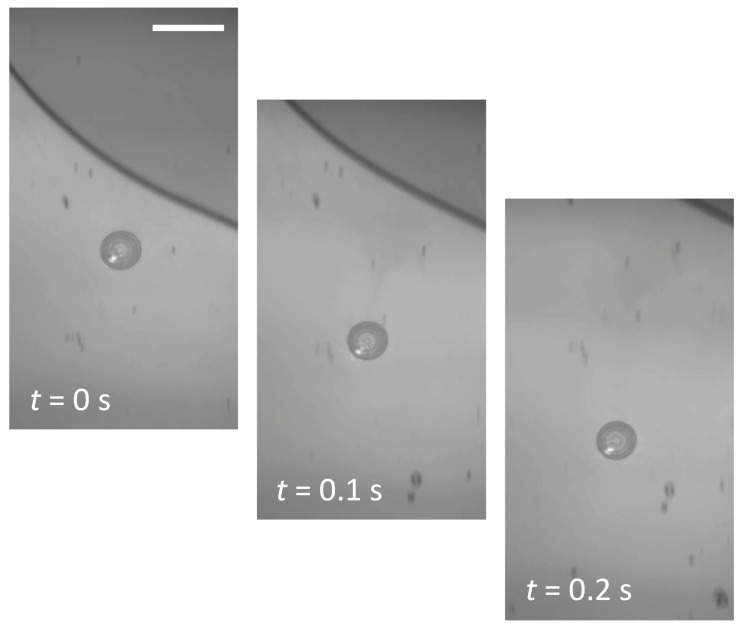
Sequential snapshots demonstrating the fast transport of a droplet by linearly translating the sample at 650 μm/s. The scale bar is 50 μm.

We next demonstrated the ability to transport the droplet to a given location. In this experiment, to observe long-distance transportation of a droplet out of the field of view, the sample was mounted on a translational stage and was linearly moved instead of the laser beam being scanned. Figure 4 shows a sequence of snapshots from the video, where a droplet with a *D*_B_ of 30 μm was dragged 130 μm in 0.2 s at a laser power of 0.48 mW (Video S4). The drag force is based on the same mechanism as droplet formation. A slight shift of the laser spot with respect to the droplet causes a temperature difference between the irradiation area and the droplet footprint area. This difference induces additional vaporization of the volatile component and an increase in the concentration of the nonvolatile (higher surface tension) component. As a result, a surface-tension gradient is generated on the droplet surface, causing solutocapillary flow toward the higher-concentration side and displacement of the droplet toward the irradiated area. This effect forces the droplet to follow the movement of the laser beam.

**Figure 5:**
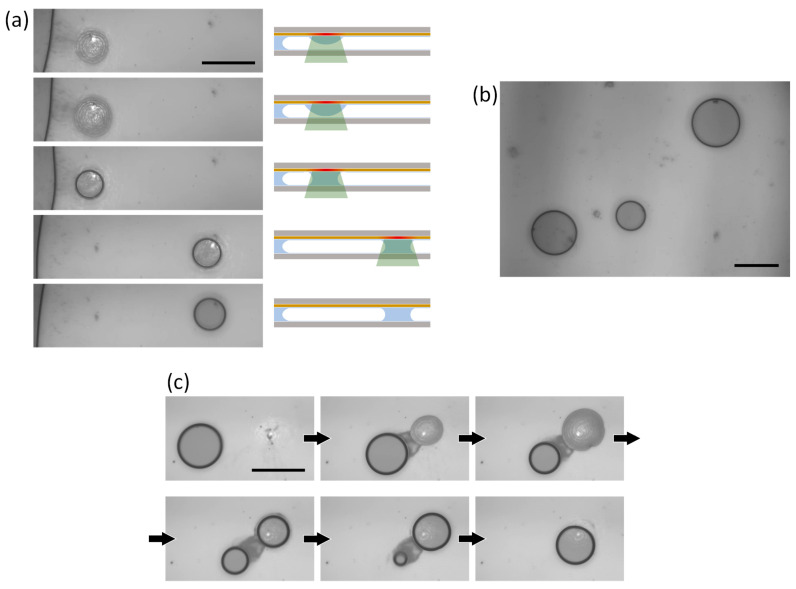
(**a**) Snapshots demonstrating the formation and transport of a droplet and the formation of a stable droplet bridge connecting the top and bottom coverslips, conducted at a laser power of 4.0 mW. The right-hand side shows a schematic side view of the slit interior. (**b**) Successive formation of multiple droplet bridges. (**c**) Snapshots demonstrating splitting and merging of droplet bridges. The scale bar is 50 μm.

In our method, the droplet can be maintained only during the laser irradiation period. Ad hoc droplet formation is useful, for instance, for cargo capture and transport. However, sustaining droplet formation without the assistance of laser irradiation is also preferable for conducting chemical and biological assays, where multiple droplet manipulations and/or massively parallel analyses of nanoscale-to-microscale objects are needed for a microreactor array. In our setup, because the separation of the two coverslips is sufficiently small, the apex of a sessile droplet can touch the bottom wall, and the droplet can grow to bridge the top and bottom walls. Once the droplet bridge is formed, it can be sustained even after the removal of laser irradiation because of the hydrophilicity of the surface of the glass coverslip. Figure 5a shows a time sequence of the growth, bridging, and dragging process at a laser power of 4.0 mW (Video S5). In this demonstration, the two coverslips were separated by ~2 μm. Bridge formation is indicated by observing the disappearance of Newton’s rings. After the droplet bridge was dragged, the laser was turned off and the droplet bridge was confirmed to be sustained. The successive creation of multiple droplet bridges was also demonstrated, as shown in Figure 5b.

Figure 5c shows the splitting and merging of a droplet bridge (Video S6). We found that dragging was possible even when the center of the laser spot was positioned outside the footprint of the droplet bridges. Solutocapillary flow outward from the edge of the droplet bridge toward the wetting film is the driving force for trapping and dragging. With further displacement of the laser-beam spot from the droplet bridge and an increase in the laser power to 4.0 mW, we could create a daughter droplet, which is equivalent to the “splitting” of droplets (upper row of Figure 5c). The mechanism behind this process is the same as that previously described for single-droplet formation. If the laser irradiation is removed at the moment of creation of a daughter droplet (bottom-left snapshot of Figure 5c), two independent droplets are stably sustained. In the recording, we continued the laser irradiation, and the mother droplet bridge was eventually completely absorbed by the daughter droplet bridge (lower row of Figure 5c), which demonstrates “merging” of droplet bridges.

## 4. Conclusions

To summarize, we demonstrated fully optical formation and transportation of sessile droplets of a binary mixed liquid (ethanol and PEG200) on an unstructured substrate with only light-absorbing ability. The mechanism is based on solutocapillary Marangoni flow in a thin wetting film. The growth rate and footprint diameter of the droplet can be controlled via modulation of the laser power. Typically, the formation time is less than 1 s, and deformation by laser power modulation at 10 Hz is possible. The fast transport of a droplet was demonstrated by moving the laser spot at a speed of 650 μm/s. To sustain the droplet without the assistance of laser irradiation, we developed a method to bridge the droplet between the top and bottom walls. Not only the formation of droplet bridges but also their splitting and merging were demonstrated.

In this study, GST was employed just as a light-absorbing material. GST undergoes a reversible change between metal-like crystalline and dielectric-like amorphous phases via thermal processes, exhibiting a huge contrast in the optical index, electronic and thermal conductivities, and hydrophilicity between its two phases. Such a distinct contrast in physical properties drastically alters the Marangoni effect [29]. As future perspective, GST can provide real-time switching and memory functionalities in the manipulation of sessile droplets [30].

One possible application of the droplet bridge is to serve as a “local reservoir”, such as supplying liquid to droplets and interacting with droplets through a wetting layer. The ability to form droplet bridges at any position and size is of great significance to increase the versatility of droplet control.

## Data Availability

Data underlying the results presented in this paper are not publicly available at this time but may be obtained from the authors upon reasonable request.

## Supplementary Materials

The following supporting information can be downloaded at https://www.mdpi.com/article/10.3390/mi14071460/s1: Video S1. Droplet formation process; Video S2. Droplet deformation process by modulating the laser power at 4 Hz; Video S3. Droplet deformation process by modulating the laser power at 10 Hz. Video S4. Transport of a droplet. Video S5. Growth, bridging, and dragging process of a droplet. Video S6. Splitting and merging of droplet bridges.
